# Editorial

**Published:** 1914-06

**Authors:** 


### EDITORIAL

The Business Office of the *Journal-Record* is 23 West Alabama Street.

The Editorial Office is 1014-15 Atlanta National Bank Building.

Address all Business Communications to *Journal-Record of Medicine*, 23 West Alabama Street.

Make remittances payable to *The Journal-Record of Medicine*.

On matters pertaining to the Editorial and Original Communications, address Edgar G. Ballenger, M. D., Atlanta, Ga.

Reprints of Original Articles will be furnished at cost price. Requests for the same should always be made in THE MANUSCRIPT.

We will present, postpaid, on request to each contributor of an original article, twenty (20) marked copies of *The Journal-Record of Medicine* containing such article.

### A BLESSING OR A CURSE.

During the last twenty years ore more numerous experiments have been made in the laboratories and among individuals hardy enough of mind and manners to submit to them, which have had in view the determination of sex at the time of conception. The labors seem at last to be approaching their fruition and we are called upon to consider what may be the effects of giving to parents or others a choice as to the sex of their offspring.

So far as cattle are concerned, we need only think of the commercial aspect, for we are unwilling to ascribe to even the most elevated of the animals under our control the mental qualities that permit of thought as to the sex value of the infant offspring. It is probable, though, that even here the continual selection in avance of a majority of one sex would eventually have a marked influence upon the breed because of the manner of transmission of hereditary qualities through the alternation of the sexes in a certain family. So far as an individual breeder is concerned, he might be profitably satisfied with his power to determine what all the young were to be, but in the long run and taking into consideration the effect of the wide spreading influence, there might have to be a clearing house to decide what was the best arrangement for all breeders, and the manager of this organization would not outdo nature very far.

But as to the genus homo. A layman after reading an account of the recent discussion of the subject at Atlantic City, exclaimed, "What a pity; if that power has been discovered, then everyone will have sons!"

He was taking things for granted and under the influence of the Scriptures was thinking of the value of the first-born where the daughters are not considered as such even if they precede their brother.

We are not ready to admit that everyone would want sons. We know of many families where the daughter is most welcome and it has been our observation that fathers, though they swell with pride to say they have brought sons to the world, yet have a great desire for the dainty sweetness a little daughter brings to the family and keeps there long after the childhood charm of a son has forever departed.

They would not always want sons. But the mothers, would they be content? It is with them that the real pride of a man child has its being, just as we are taught that it is with the preponderance of strength for the time being of the female generative element there comes a strong force determining the male sex of the child. The mothers must have something to say.

Again, something unusual and categorical must be done at the time of conception in order to carry out the principles that will decide the child's sex.

Thus we have the father, the mother and the method as determined and possibly directed by some one else. Is this a blessing or a curse? Leave aside all old-fashioned notions as to the sacredness of the emotions that come to the truly wedded with their love; admit if you please that because the child is eventually to take its place in the community, there should be no unviolable privacy in the parental copulative act or the things that lead up to it; take the scientific viewpoint and do everything for the best, all society considered.

Then, will it be a blessing or a curse? Consider the longing of one parent for a daughter and the other for a son. Who is to decide which shall prevail? The doctor or public censor, perhaps. But when the decision is made, who will carry it out? And who will heal and soothe the heart burnings of the disappointed one? Let us suppose a mother wanting a son and yet having to submit all the weary months of a trying pregnancy to the burden of carrying an unwelcome daughter forced upon her by the new meth[illegible]ls. We are not worrying over prenatal impressions nor those that a distraught mother might by some miracle convey to her child, but we are certain that a nervous and unhappy woman doesn't make as good a mother either before or after the birth of her child as does the happy and contented one.

However, someone has said that man must have sublime faith in his wife bcause it is she alone who knows who is the father of her child. This faith is justified thanks be to the character of the womanhood our civilization has developed. But will she continue to do as he says when she wants her own children her own way? No matter what the rules laid down by the interloping scientist, it is she eventually who will determine whether they are carried out and it is she that can mislead everyone else regarding them. The child that comes contrary to the expectation will be a mistake of the system to everyone but her and her friends the other mothers who will have their way. Is it a blessing or a course to induce deceit into the family concerning its most sacred function? As the generation develops and the children grow to an age of understanding, the they likely to have their respect for their parents increaesd by the aspect of sex determination and the machine-like quality of their genesis?

There may be use for sex determination among ruling families whom we encourage to breed themselves in certain strains for certain purposes,, mainly those of show, but among the peoplt we depend upon for the honor and integrity of the nations, we expect to find a continuance of that privacy and delicacy of the consummation of the marital life which gives the character of holiness to recognized parenthood.

